# In Vivo Serial MR Imaging of Magnetically Labeled Endothelial Progenitor Cells Homing to the Endothelium Injured Artery in Mice

**DOI:** 10.1371/journal.pone.0020790

**Published:** 2011-06-24

**Authors:** Jun Chen, Zhen-Yu Jia, Zhan-Long Ma, Yuan-Yuan Wang, Gao-Jun Teng

**Affiliations:** Jiangsu Key Laboratory of Molecular Imaging and Functional Imaging, Department of Radiology, Zhongda Hospital, Southeast University, Nanjing, China; National Institute of Health, United States of America

## Abstract

**Background:**

Emerging evidence of histopathological analyses suggests that endothelial progenitor cells (EPCs) play an important role in vascular diseases. Neointimal hyperplasia can be reduced by intravenous transfusion of EPCs after vascular injury in mice. Therefore, it would be advantageous to develop an in vivo technique that can explore the temporal and spatial migration of EPCs homing to the damaged endothelium noninvasively.

**Methodology/Principal Findings:**

The left carotid common artery (LCCA) was injured by removal of endothelium with a flexible wire in Kunming mice. EPCs were collected by in vitro culture of spleen-derived mouse mononuclear cells (MNCs). EPCs labeling was carried out in vitro using Fe_2_O_3_-poly-L-lysine (Fe_2_O_3_-PLL). In vivo serial MR imaging was performed to follow-up the injured artery at different time points after intravenous transfusion of EPCs. Vessel wall areas of injured artery were computed on T_2_WI. Larger MR signal voids of vessel wall on T_2_WI was revealed in all 6 mice of the labeled EPC transfusion group 15 days after LCCA injury, and it was found only in 1 mouse in the unlabeled EPC transfusion group (p = 0.015). Quantitative analyses of vessel wall areas on T_2_WI showed that the vessel wall areas of labeled EPC transfusion group were less than those of unlabeled EPC transfusion group and control group fifteen days after artery injury (p<0.05). Histopathological analyses confirmed accumulation and distribution of transfused EPCs at the injury site of LCCA.

**Conclusions/Significance:**

These data indicate that MR imaging might be used as an in vivo method for the tracking of EPCs homing to the endothelium injured artery.

## Introduction

Percutaneous transluminal angioplasty (PTA) and stenting are increasingly being used for the treatment of atherosclerotic stenosis. However, neointimal hyperplasia, restenosis and late stent thrombosis remain a source of frustration [1]–[3]. Endothelial dysfunction is considered to be the initiation of arteriosclerosis process, and endothelial damage is a major cause of neointimal hyperplasia and restenosis after endoluminal procedure [4], [5]. Recently, emerging evidence suggests that endothelial progenitor cells (EPCs) play an important role in the prevention of arteriosclerosis, reduction of neointima formation and attenuation of restenosis by promoting re-endothelialization [6]–[8].

By histopathological analyses, intravenous transfusion of EPCs homing to the site of vascular injury and resulting in an enhanced re-endothelialization associated with decreased neointima formation was demonstrated [7]. The ability to inhibit neointimal hyperplasia can be further enhanced by genetically engineered EPCs [9]. However, it would be advantageous to develop an in vivo technique that can monitor the transplanted cells. Especially it might be extremely useful for the potential clinical application in the future.

Several studies have confirmed the applicability of in vivo MR imaging in the observation of artery injury and arteriosclerosis in different animal models [10]–[12]. With superparamagnetic iron oxide (SPIO)-based method, some researchers have initially investigated the feasibility of MR techniques for in vivo cellular imaging in animal models of different diseases [13]–[17]. A study by Shapiro et al showed that in vivo MR can even detect a single group of cells homing to tissue following cell labeling and transplantation [18]. We postulate that in vivo MR imaging may be a potential new technique to explore the temporal and spatial migration of EPCs homing to the damaged endothelium noninvasively.

## Results

### Animal Model and EPCs

Splenectomy and carotid artery injury were successfully performed in all of the mice.

**Figure 1:**
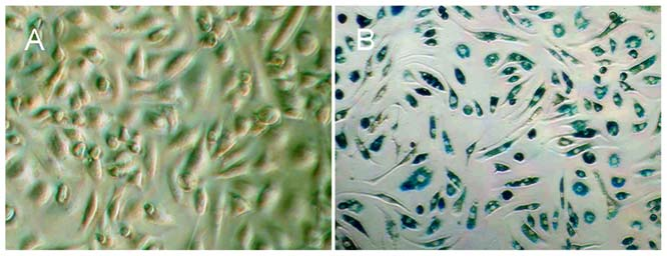
spleen-derived EPCs. EPCs showed endothelium-like cobblestone morphology with characteristic formation of clusters (A). Blue particles are clearly visible in almost every labeled cell with Prussian blue staining (B). (Magnification ×200).

**Figure 2:**
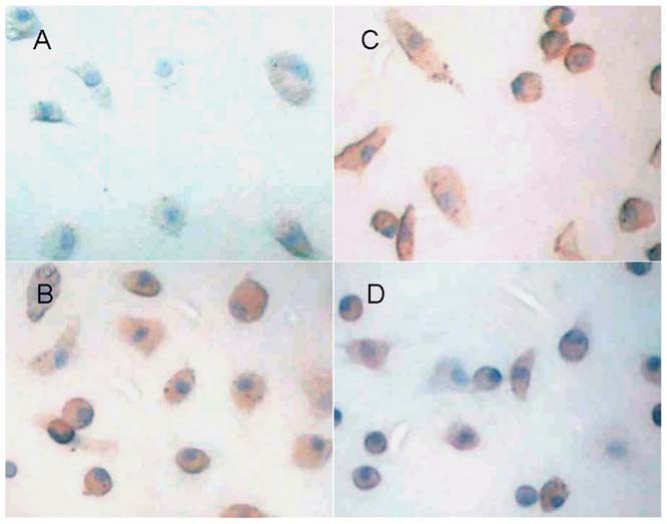
Immunostaining of spleen-derived EPCs. 7 days after in vitro differentiation, spleen-derived EPCs showed expression of CD31 (B), CD34 (C) and vWF (D) with the cytoplasm stained in brown. Photo A is the control. Hematoxylin-stained nuclei were shown in blue. (Magnification ×400).

**Figure 3:**
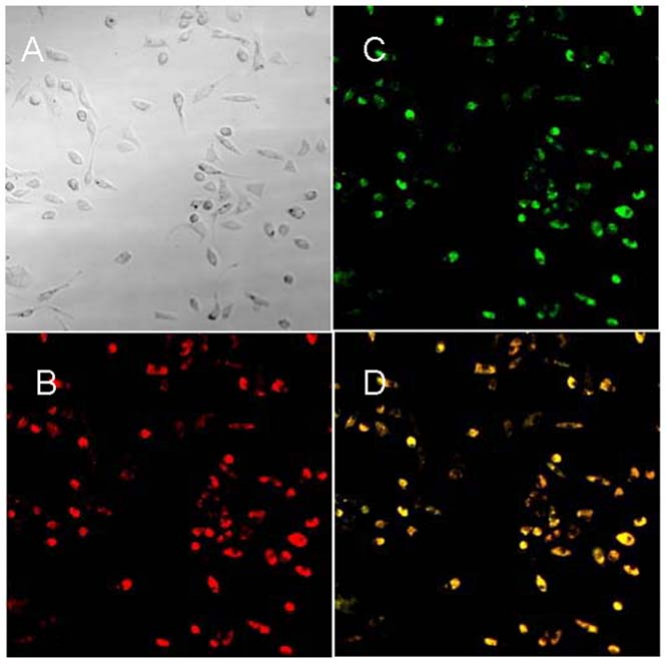
Endothelial cell characteristics. After 7 days culture (A), cells taken up DiI-ac-LDL were red fluorescence (B), bound to FITC-UEA-I displayed green fluorescence (C), and the double-stained cells showed yellow fluorescence in cytoplasm (D). (Magnification ×200).

In vitro cultured spleen-derived MNCs showed the typical “cobblestone” appearance of endothelial cells (Figure 1A). The cells showed expression of CD31, CD34 and vWF with the cytoplasm stained in brown, confirming that the cells had typical characteristics of EPCs after in vitro culture with medium EGM-2 (Figure 2). Cultured EPCs that had taken up DiI-ac-LDL displayed red fluorescent aggregates (Figure 3B). Cultured EPCs bound to the endothelial cell specific FITC-UEA I were green fluorescent aggregates (Figure 3C). EPCs that had taken both DiI-ac-LDL and FITC-UEA I displayed yellow fluorescence in the cytoplasm (Figure 3D). Blue particles could be seen in almost every Fe2O3-PLL labeled cell in Prussian blue staining (Figure 1B).

### In Vivo MR Imaging

**Figure 4:**
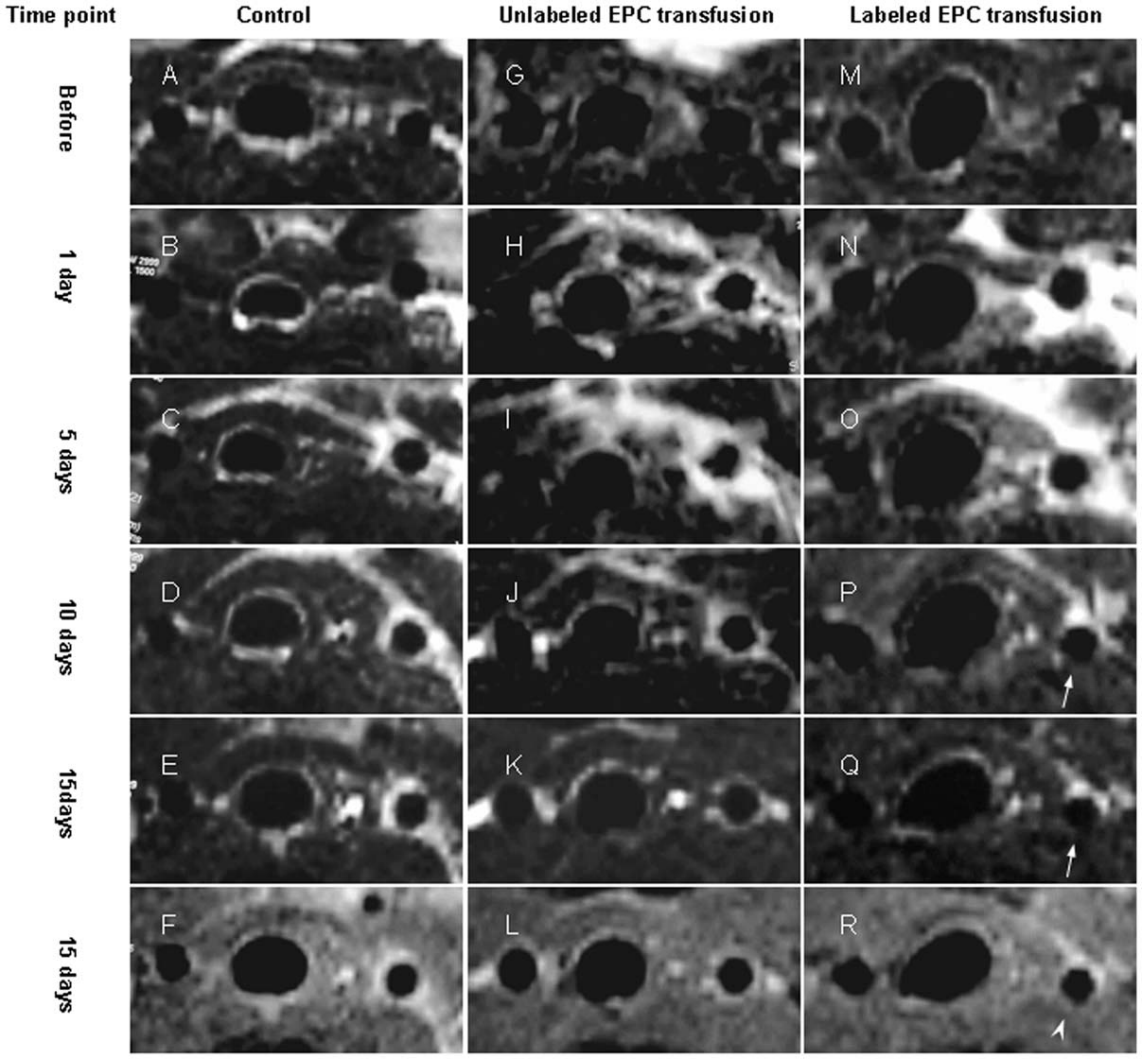
In vivo serial MR imaging. Figure 4A–4E, Figure 4G–4K and Figure 4M–4Q indicated T_2_WI. Figure 4F, 4L, and 4R indicated PDWI. Before artery injury, LCCA appeared normal on MR image without wall thickening and stenosis. After artery injury, wall thickening and stenosis of LCCA was obviously observed. Large MR signal voids of vessel wall was revealed on T_2_WI at 10 days and 15 days after artery injury (Arrow, Figure 4P, 4Q). However, the vessel wall was visible on PDWI (arrow head, Figure 4R).

The signal voids of lumen, wall thickening of injured artery and exudation around injured artery were clearly observed on serial PDWI in company with T_1_WI and T_2_WI at different time points (Figure 4). Before artery injury, LCCA (left carotid common artery) looked normal (as ring) on MR image without wall thickening and stenosis. One day after artery injury, deformation of LCCA was obvious on all sequences of MR image. Evident exudation due to inflammatory reaction around LCCA was observed on T_2_WI within 10 days after artery injury. RCCA (right carotid common artery) looked normal at MR image, without wall thickening and stenosis.

Larger MR signal voids of vessel wall on T_2_WI was revealed in all 6 mice in the labeled EPC transfusion group at 15 days after LCCA injury (Figure 4P, 4Q), while it was found only in 1 mouse in the unlabeled EPC transfusion group (p = 0.015), which indicated that the SPIO particles may be contributed to the large MR signal voids of vessel wall due to their properties of T_2_WI sensitivities.

**Figure 5:**
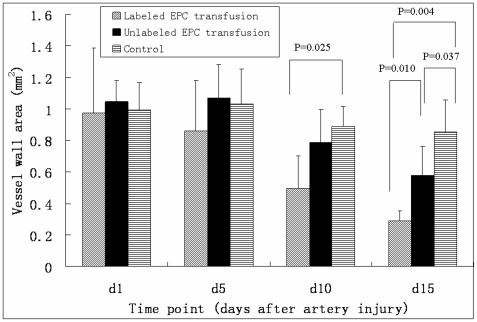
Vessel wall area (mm^2^) of LCCA measured on T_2_WI at different time points. There was no significant difference of vessel wall area among the three groups 1 day and 5 days after artery injury. However, vessel wall area of labeled EPC transfusion group was significantly less than that of unlabeled EPC transfusion group and control group at 15 days after artery injury. Vessel wall areas of EPCs transfusion group were significantly less than that of non-EPC transfusion group at 15 days after artery injury.

Vessel wall areas of LCCA were quantitatively analyzed on T_2_WI at different time points (Figure 5). There were significant differences of vessel wall area among the three groups 10 day (χ^2^ = 6.678, p = 0.035) and 15 days (χ^2^ = 12.433, p = 0.002) after artery injury. The vessel wall area of labeled EPC transfusion group were less than that of unlabeled EPC transfusion group and control group at 15 days after arterial injury (0.289±0.065 mm^2^ vs. 0.578±0.187 mm^2^,u = 2.000, p = 0.010; 0.289±0.065 mm^2^ vs. 0.855±0.202 mm^2^, u = 0.000, p = 0.004). Also, there was a significant difference of vessel wall areas between unlabeled EPC transfusion group and control group 15 days after artery injury (0.578±0.187 mm^2^ vs. 0.855±0.202 mm^2^, u = 5.000, p = 0.037).

### Histopathological Findings

**Figure 6:**
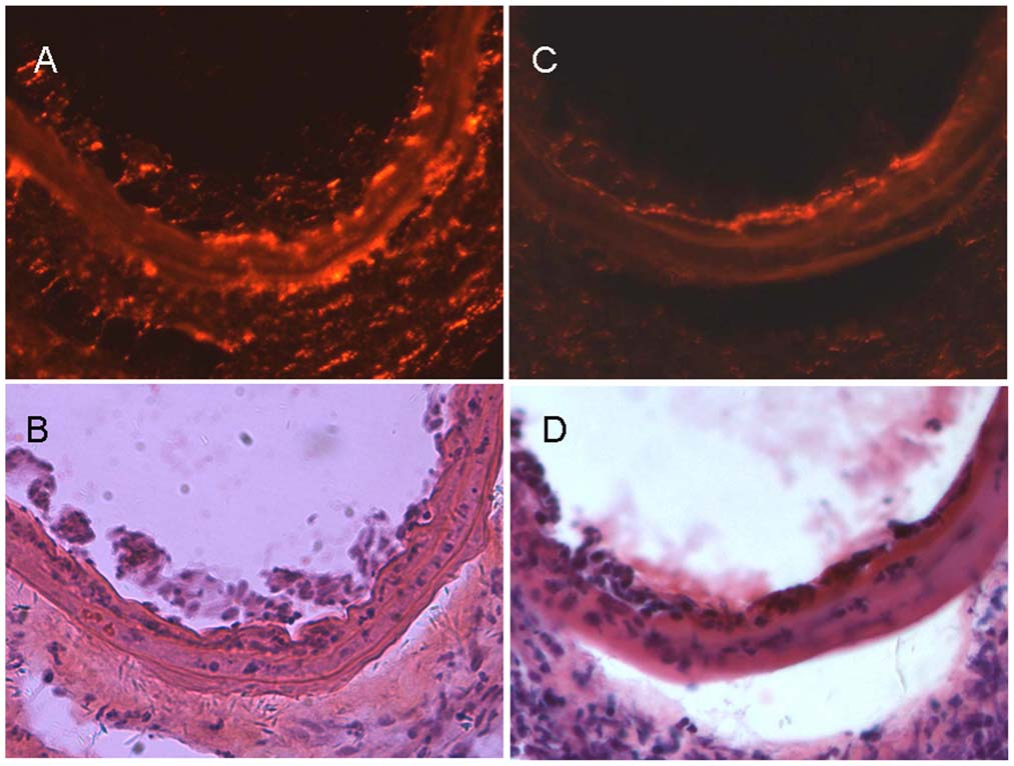
Transfused EPCs contribute to reendothelialization. Laser scanning confocal microscopic analysis (6A, 6C) and H&E staining (6B, 6D) demonstrated accumulation of transfused Dil-Ac-LDL-labeled EPCs at the injury site 5 days (6A, 6B) and 10 days (6C, 6D) after endothelium damage of LCCA. (Magnification ×200).

**Figure 7:**
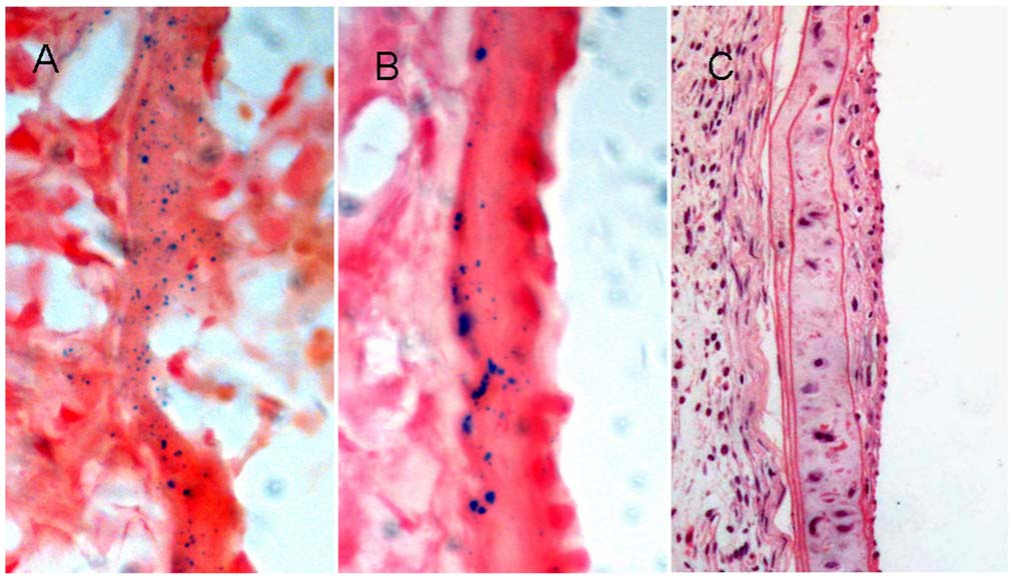
Histopathological analysis. Prussian blue staining showed blue particles of Fe_2_O_3_-PLL labeled EPCs distributing in subendothelium (Figure 7A, 7B indicated 5, 10 days after endothelium injury respectively). Figure 7C showed crescentic neointima of injured artery in 2 animals of non-EPC transfusion group. (Magnification ×200).

Laser scanning confocal microscopic analysis (Figure 6A, 6C) and H&E staining (Figure 6B, 6D) demonstrated accumulation of transfused Dil-Ac-LDL-labeled EPCs at the injury site after endothelium damage of LCCA. Prussian blue staining confirmed blue particles of Fe_2_O_3_-PLL labeled EPCs distributing in the injured endothelium (Figure 7A,7B). Intimal hyperplasia of LCCA was visible in all the animals of the three groups, and crescentic neointima of LCCA was confirmed in two animals of control group (Figure 7C). No intimal hyperplasia of right common carotid artery (RCCA) was found.

## Discussion

EPC was isolated primarily in 1997 by Asahara et al [19], and has been extensively studied in cardiovascular diseases [20], [21]. MR imaging holds several advantages for tracking cells in vivo, such as high spatial resolution, high temporal resolution, high penetration depth, etc. In vivo MR imaging therefore appears promising for dynamically monitoring cell migration after transplantation. For the tracking of EPCs using MR imaging, in vitro labeling of the cells is essential. As a MR imaging contrast agent, SPIO which creates hypointense signals on T_2_WI has been widely used in cell labeling [22]–[24]. The administration of ferumoxides-poly-L-lysine labeled human stem cells appeared not to have a significant effect on hematology, blood chemistry indicators, or organ function in rats [25]. It was found that the iron in endosome was not released into the cytoplasm as a free radical form, which would be toxic to the cells [26]. After many iron particles were injected into single-cell embryos, the embryos developed normally, which indicates that the iron particles may be nontoxical to embryos development [27]. Both peripheral blood derived EPCs and spleen derived EPCs can be effectively labeled using Fe_2_O_3_-PLL with little influence on cells' biological properties [23], [28]. The spleen derived EPCs labeled with Fe_2_O_3_-PLL can even be detected as a single group of cells on in vitro MR imaging using high field strength 7T MR scanner [28]. Based on these studies, we used Fe_2_O_3_-PLL to label spleen derived EPCs in vitro for MR imaging in the current study.

The imaging artifact caused by iron oxide particles may be several times larger than the labeled cell [29]. As transfused EPCs might attach to the injured endothelium and contribute to the re-endothelialization, transfusion of Fe_2_O_3_-PLL labeled EPCs led to large signal voids of vessel wall on T_2_WI presumably due to SPIO induced signal intense loss in the present study (Figure 4, 5). The accumulation of transfused Dil-Ac-LDL-labeled EPCs at the site of injured endothelium confirmed by Laser scanning confocal microscopic observation and histopathological analysis validated spleen-derived EPCs transfused intravenously homing to the injured artery (Figure 6). The blue particles of Fe_2_O_3_-PLL labeled EPCs distributing in the injured endothelium demonstrated by Prussian blue staining showed that the SPIO are efficiently labeled with EPCs as the protocol (Figure 7). Our study shows encouraging results that in vivo cellular MR imaging may be a potential method for the tracking of EPCs transfused intravenously. However, larger MR signal voids of vessel wall was also found in 1 mouse in the unlabeled EPC transfusion group at 15 days after LCCA injury. We presume that a potential reason for this to happen was mismatching of MR imagings at different time points.

It was reported that endosomal iron nanoparticles could be detected after seven weeks in nondividing human mesenchymal stem cells, and intracellular iron had disappeared by five to eight divisions in rapidly dividing cells [30]. In the study by Shapiro et al, a single group of particles injected into single cell-stage mouse embryos could be detected at embryonic day 11.5, demonstrating that daughter cells carry a single group of particles can still be detected even after many cell divisions [27]. The strength of the present study is that individual carotid artery was followed serially by in vivo MR imaging of T_2_WI after EPC transfusion. In vivo quantitative analysis on MR imaging is also a strength feature in this study. The approach provides in vivo insights into the natural history of SPIO-labeled EPCs in endothelial injury. The data indicated that vessel wall area on T_2_WI in SPIO-labeled EPC transfusion group was less than those in unlabeled EPC transfusion group and control group at fifteen days after artery injury. Vessel wall area measured on T_2_WI not only accounts for signal intense loss induced by SPIO but also indicates neointimal hyperplasia indirectly. The vessel wall area of unlabeled EPC transfusion group was not as much as that of control group fifteen days after artery injury in the present experiment (Figure 5). The result was similar to previous studies which suggested that EPCs may contribute to endothelial regeneration thereby limiting neointimal hyperplasia [7], [31]. Our quantitative data on MR imaging accurately showed the homing of EPCs and their role on the repair of artery injury.

To what extents the transfused EPCs die and release SPIO particles may pose a problem. It was considered that any SPIO released into the blood would be taken up by the Kupffer cells in the liver and in the reticuloendothelial system in the spleen [32], [33]. Although spleen was dissected to prolong the circulating time of EPCs in the peripheral circulation and allow homing of transfused spleen-derived EPCs to the injured artery rather than to the spleen, we did find SPIO in the liver of mice in the current study (data not presented). Therefore, it is unlikely that the iron particles released from labeled EPCs would be a problem in the observation of endothelium injury in carotid artery. The correlationship between MR imaging and histopathology has been demonstrated by several studies on neointimal hyperplasia and atherosclerotic plaque in different animal models [10]–[12], however, we can not answer the correlationship between the numbers of Fe_2_O_3_-PLL labeled EPCs transfused intravenously and signal intense loss of vessel wall based on the limited data, which is one of the major limitations of the current study.

In conclusion, initial evidence from our study implies that in vivo MR imaging can noninvasively and dynamically depict systemically administered EPCs homing to injured endothelium on the basis of cell labeling with SPIO in mice. Moreover, in vivo quantitative analysis for signal intense loss of vessel wall on T_2_WI induced by SPIO labeled EPCs is feasible on MR imaging. We believe that such an experimental platform of in vivo cellular MR imaging would open the door to understand treatment efficacy of transplanted cells in arterial diseases.

## Materials and Methods

### Animal Model

All animal procedures were carried out in accordance with approvals and the guidelines of Institutional Animal Care and Use Committee (IACUC) of Medical School of Southeast University(approval ID: SYXK-2007.2122). A total of 22 male Kunming mice (Animal Center of Southeast University, Nanjing, China), 18–20 weeks of age weighted at 35.0–45.0 g, were recruited, and fed with conventional diet.

Animal model was established based on modifications of previously described methods [34], [31]. Animals were anesthetized by intraperitoneal injection of 80 mg/kg Pentobarbital (Shanghai Chemical Reagent Co., Shanghai, China). The spleen was dissected through a lateral incision of the left abdomen. Carotid artery injury was carried out seven days after spleenectomy. The bifurcation of the left common carotid artery (LCCA) was exposed under a dissecting microscope (ZOOM645S, Jiangnan Yucheng, China). Two ligatures were placed proximally and distally around the external carotid artery. The distal ligature was then tied off. After temporary occlusion of the internal and common carotid artery, an incision hole was made between the ligatures of the external carotid artery. A curved flexible wire with 0.27 mm in diameter was introduced into the LCCA via the incision. The wire was passed along the LCCA in a rotating manner for three times. After removal of the wire, the proximal ligature of the external carotid artery was tied off. Normal blood flow was reassured, and the skin was closed with single sutures using 6/0 silk.

### Spleen-Derived EPCs and Their Characteristics

Based on modifications of previously described methods [7], [31], spleens from Kuming mice (Animal Center of Southeast University, Nanjing, China) were explanted and mechanically minced. The cells suspended in 6 ml PBS were centrifuged in a density-gradient centrifuge Histo-Paque 1077 (Sigma-Aldrich, St. Louis, MO) at 400 g for 30 min. A sediment layer of mononuclear cells (MNCs) was collected, washed twice with PBS, suspended in microvascular growth medium-2 (EGM-2; Cambrex, Walkersville, MD) and adjusted to a concentration of 2×10^6^/ml. The mononuclear cells were placed in 6-well or 24-well culture plate (Corning Inc., Corning, NY) and cultured at 37°C under 100% humidity and 5% CO_2_. 3 days after, the suspended cells were removed and the cells adhering to the plate wall were fed every 3 days with fresh culture medium.

After 7 days in culture, spleen-derived EPCs in 4 wells of a 24-well culture plate were extensively washed, and fixed in ice-cold 4% paraformaldehyde for 20 min. Immunocytochemistry was performed using antibodies against CD31, CD34 (Wuhan Boster Biological Technology, LTD. Wuhan, China) and von Willebrand factor (vWF) ( Zhongshan Goldenbridge Biotechnology Co., LTD. Beijing, China) according to standard protocols.

Spleen-derived EPCs that had been cultured for 7 days were incubated in one well of a 6-well culture plate with 2.0 mL culture medium containing 10 µg/ml DiI-Ac-LDL (1, 1′-dioctadecyl-3, 3, 3′, 3′- tertamethylindo-carbocyanine -labeled acetylated low-density lipoprotein) (Biomedical Technologies Inc., Stoughton, MA) at 37°C for 6 hours. Then the cells were washed with PBS twice and fixed in 4% paraformaldehyde for 20 min. The fixed cells were then incubated with 1 mL (10 µg/ml) FITC-UEA I (Vector Laboratories Inc., Burlingame, CA) at 37°C for 1 hour. EPCs were observed under a laser scanning confocal microscope (LSM 510; Zeiss Co. Ltd., Oberkochen, Germany). The adherent cells that stained positive with both FITC-UEA I and DiI-Ac-LDL were indicated to be differentiating endothelial cells.

### Cell Labeling

The preparation of superparamagnetic particles Fe_2_O_3_-poly-L-lysine (Fe_2_O_3_-PLL) and cell labeling were carried out based on previously described methods [22]. Spleen-derived EPCs that had been cultured for 4–6 days were incubated in one well of a 6-well culture plate with 2.0 mL culture medium containing 20 µg/ml Fe_2_O_3_-PLL for 12 hours. After incubation, the EPCs were washed with phosphate-buffered saline 3 times to remove excess Fe_2_O_3_-PLL. For Prussian blue staining, the cells were incubated with 2% potassium ferrocyanide (Shantou Xilong Chemical Inc., Guangdong, China) in 6% hydrochloric acid (HCl) and then counterstained with nuclear fast red, which reveals the cells' profile and intracellular iron particles.

### Animal Groups and Cell Transfusion

After 5–7 days in culture, cell transfusion was carried out by intravenous tail vein injection directly after induction of arterial injury and again 24 hours later. The mice (n = 22) were randomly divided into four groups. In group one, the mice received 1×10^6^/0.5 ml Fe_2_O_3_-PLL labeled EPCs (labeled EPC transfusion group, n = 6). In group two, the mice received 1×10^6^/0.5 ml unlabeled EPCs (unlabeled EPC transfusion group, n = 6). In group three, the mice received a corresponding amount of phosphate buffered solution (PBS) (control group, n = 6). In group four, two mice received 1×10^6^/0.5 ml Fe_2_O_3_-PLL labeled EPCs, and two mice received 1×10^6^/0.5 ml DiI-Ac-LDL labeled EPCs. The mice were observed with in vivo serial MR imaging and sacrificed after the last MR imaging in group one, group two and group three (n = 18, 6 mice in each group). The mice of group four were sacrificed at 5 days (n = 2) and 10 days (n = 2) respectively after artery injury for histopathological analysis of transplanted DiI-Ac-LDL labeled EPCs and iron particles in vessel wall of LCCA.

### In Vivo MR Imaging

In vivo MR imaging of the injured artery was performed on a 7T Micro-MR imaging system (Bruker PharmaScan micro-MR, Germany) with a 35-mm birdcage coil at different time points (before, 1, 5, 10 and 15 days after artery injury).

The mice were positioned in the prone position and a pressure-sensitive pad for respiratory gating was placed on the abdomen. The mice were anesthetized with 1.0% to 1.5% isoflurane (Shandong Keyuan Pharmaceuticals Company, Jinan, China) with air and oxygen mixed at a 3∶1 ratio. We regulated the flow of isoflurane to maintain the respiratory rate at approximately 60–80 breaths per minute. The respiratory signals were used to synchronize image acquisition to reduce imaging artifacts due to respiratory motions.

We acquired T_1_-, T_2_-, and proton density-weighted images (T_1_WI, T_2_WI and PDWI, TR/TE 1300.0/7.5, 2535.9/65.0, and 2535.9/13.0 msec, respectively) using rapid acquisition with relaxation enhancement sequence (T_1_WI) and multi slice multi echo sequence (T_2_WI and PDWI). The slice thickness was 0.6 mm, the field of view was 3.0×3.0 cm^2^, the matrix size was 256×256, and the resolution was 117×117×600 µm^3^. Two signal averages were used in T_1_WI, and one signal average was used in T_2_WI and PDWI.

Source images were reconstructed and analyzed using the scanner console (Paravision; Bruker Biospin, Billerica, MA, USA). Vessel wall area was computed based on the cross section of image with the most significant vessel wall thickening and lumen narrowing. At different time points of MR imaging, the same section LCCA was analyzed in each mouse. The bifurcation of common carotid artery was used as a marker to ensure the same slice of LCCA was analyzed in each mouse at different time points of MR imaging and histological slides.

### Histopathological Analysis

Animals to be sacrificed were anesthetized by intraperitoneal injection of 80 mg/kg Pentobarbital (Shanghai Chemical Reagent Co., Shanghai, China). In situ perfusion fixation was achieved with injecting 4% phosphate-buffered paraformaldehyde into the left ventricle. The carotid arteries were excised. Perfusion-fixed carotid arteries were embedded in Tissue Tek OCT embedding medium (Sakura, Fine technical Co., Ltd., Tokyo, Japan), snap-frozen, and stored at −80°C. The specimens were sectioned into slices of 6–10 µm in thickness for laser scanning confocal microscopic analysis (LSM 510; Zeiss Co. Ltd., Oberkochen, Germany) to observe DiI-Ac-LDL, for hematoxylin-eosin staining to display tissue morphology and for Prussian blue staining to identify iron particles.

### Statistical Analysis

All obtained quantitative data were expressed as means ± standard deviation (SD). Statistical analyses were performed with the SPSS software (SPSS for Windows, version 11.0, 2001; SPSS, Chicago, IL). Test for several independent samples (Kruskal-Wallis H) was used to test the difference of vessel wall area among groups. The significances of vessel wall area between two groups were determined by test for two independent samples (Mann-Whitney U). Fisher's exact test was employed to determine the significance of the cases with large MR signal voids of vessel wall between two groups. P values<0.05 were considered statistically significant.
